# Supplementary figures and images for: Increased collective migration correlates with germline stem cell competition in a basal chordate
Source: PLoS One. 2023 Oct 30;18(10):e0291104. doi: 10.1371/journal.pone.0291104 (PMC10615308; doi:10.1371/journal.pone.0291104)

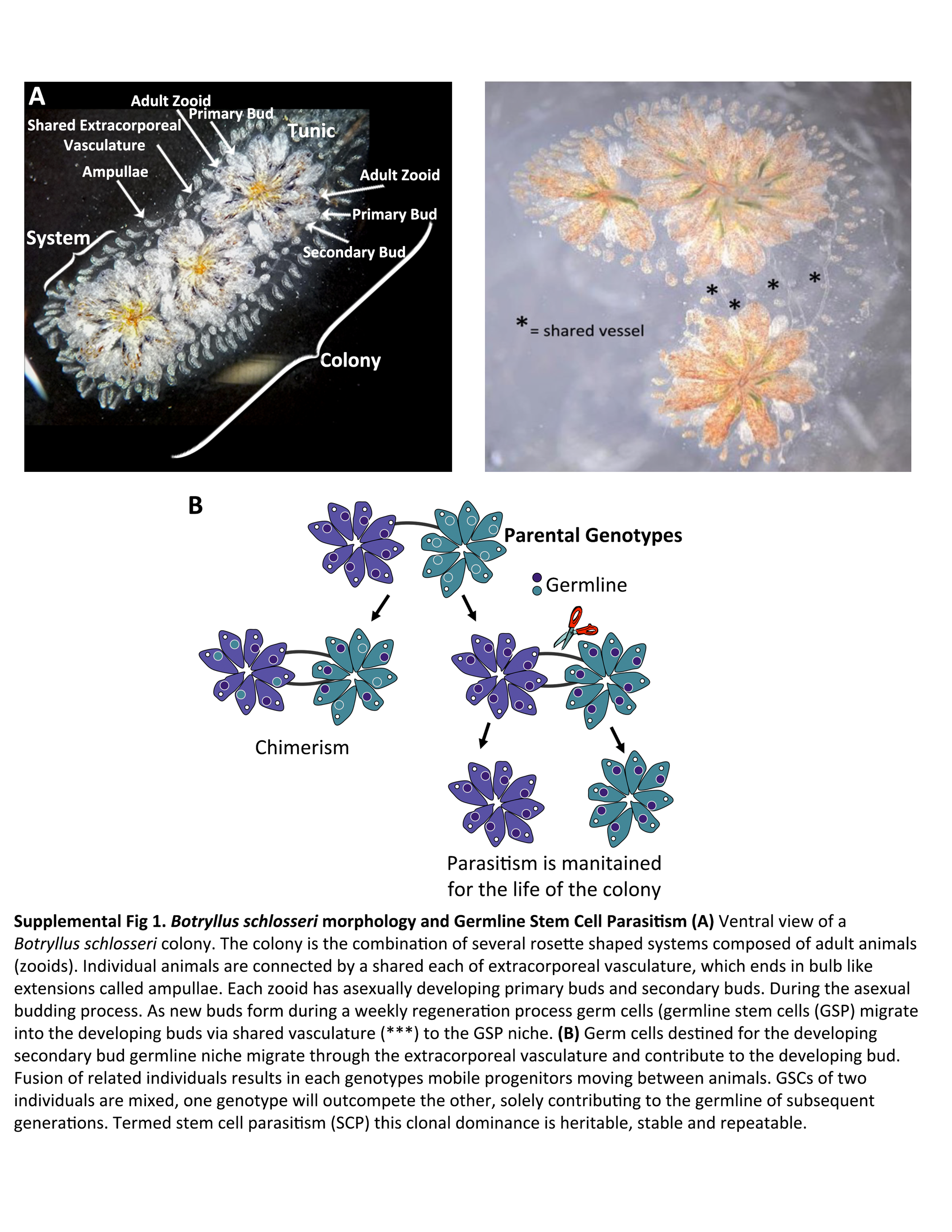

Supplement: S1 Fig — (A). Ventral view of a Botryllus schlosseri colony. The colony is the combination of several rosette shaped systems composed of adult animals (zooids). Individual animals are connected by a shared extracorporeal vasculature, which ends in bulb like extensions called ampullae. Each zooid has asexually developing primary buds and secondary buds. As new buds form during a weekly regeneration process, germline stem cells (GSCs) migrate into the developing buds via the shared vasculature (***) to the GSC niche. (B) Germ cells destined for the developing secondary bud germline niche migrate through the extracorporeal vasculature and contribute to the developing bud. Fusion of related individuals results in each genotypes mobile progenitors moving between animals. When GSCs of two individuals are mixed, one genotype will outcompete the other, solely contribution to the germline of subsequent generations. Termed stem cell parasitism (SCP), this clonal dominance is heritable, stable and repeatable, even if the vascular connection is severed. (TIF) [file pone.0291104.s001.tif]

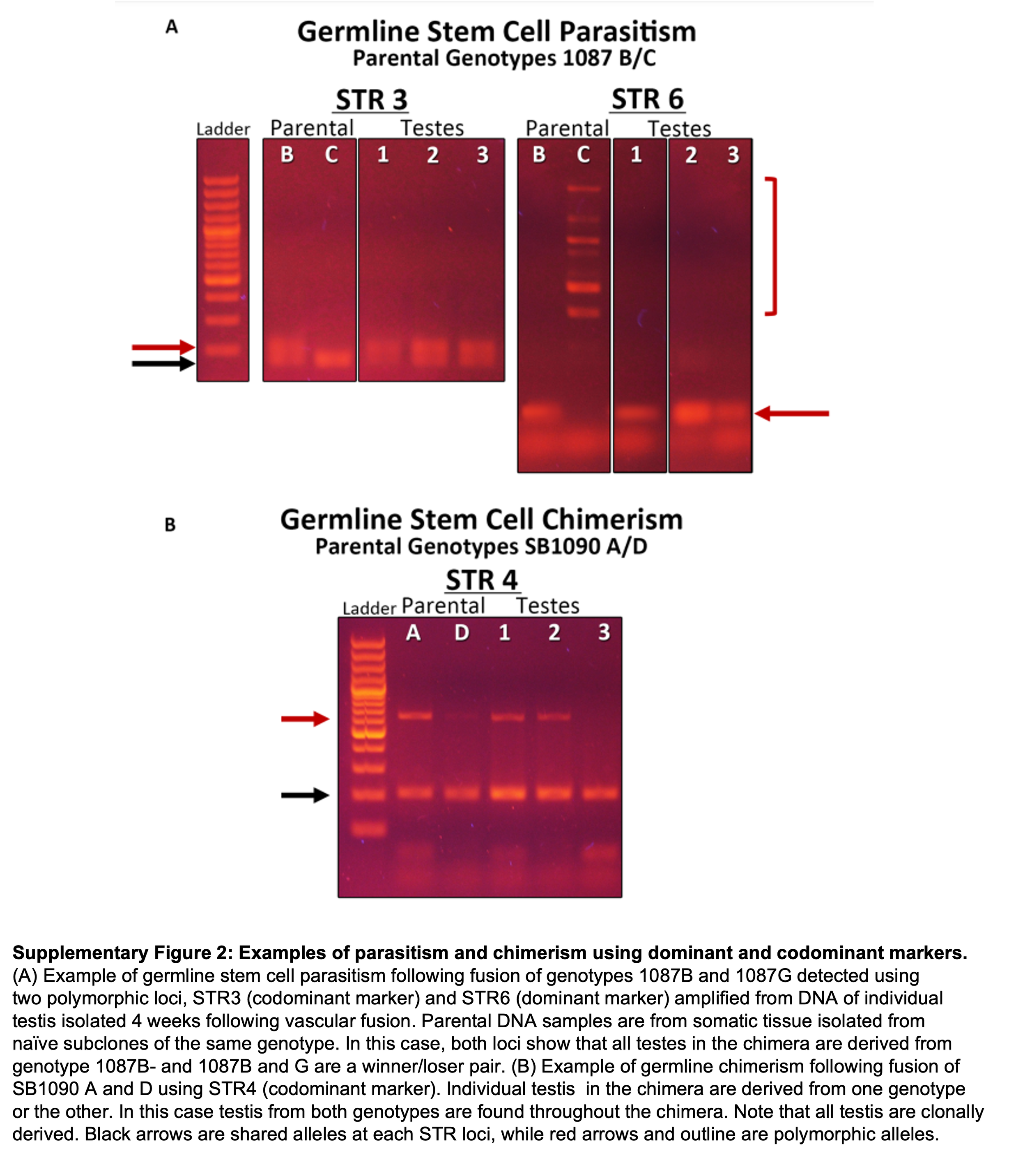

Supplement: S2 Fig — (A) Example of germline stem cell parasitism following fusion of genotypes 1087B and 1087G detected using two polymorphic loci, STR3 (codominant marker) and STR6 (dominant marker) amplified from DNA of individual testis isolated 4 weeks following vascular fusion. Parental DNA samples are from somatic tissue isolated from naïve subclones of the same genotype. In this case, both loci show that all testis in the chimera are derived from genotype 1087B. Thus, 1087 B and G are a winner/loser pair. (B) Example of germline chimerism following fusion of SB1090A and SB1090D using STR4 (codominant marker). In this case, individual testis in the chimera are derived from one genotype or the other. Note that all testis are clonally derived. Black arrows are shared alleles at each STR loci, while red arrows and outline are polymorphic alleles. (TIF) [file pone.0291104.s002.tif]
